# Molecular and functional characterization of two isoforms of chalcone synthase and their expression analysis in relation to flavonoid constituents in *Grewia asiatica* L

**DOI:** 10.1371/journal.pone.0179155

**Published:** 2017-06-29

**Authors:** Tareq A. Wani, Shahzad A. Pandith, Ajai P. Gupta, Suresh Chandra, Namrata Sharma, Surrinder K. Lattoo

**Affiliations:** 1 Genetic Resources and Agrotechnology Division, CSIR-Indian Institute of Integrative Medicine, Jammu Tawi, India; 2 Plant Biotechnology Division, CSIR-Indian Institute of Integrative Medicine, Jammu Tawi, India; 3 Quality Control and Quality Assurance Division, CSIR-Indian Institute of Integrative Medicine, Jammu Tawi, India; 4 Department of Botany, University of Jammu, Jammu Tawi, India; Southern Crop Protection and Food Research Centre, CANADA

## Abstract

Chalcone synthase constitutes a functionally diverse gene family producing wide range of flavonoids by catalyzing the initial step of the phenylpropanoid pathway. There is a pivotal role of flavonoids in pollen function as they are imperative for pollen maturation and pollen tube growth during sexual reproduction in flowering plants. Here we focused on medicinally important fruit-bearing shrub *Grewia asiatica*. It is a rich repository of flavonoids. The fruits are highly acclaimed for various putative health benefits. Despite its importance, full commercial exploitation is hampered due to two drawbacks which include short shelf life of its fruits and larger seed volume. To circumvent these constraints, seed abortion is one of the viable options. Molecular interventions tested in a number of economic crops have been to impair male reproductive function by disrupting the chalcone synthase (CHS) gene activity. Against this backdrop the aim of the present study included cloning and characterization of two full-length cDNA clones of *Ga*CHS isoforms from the CHS multigene family. These included *Ga*CHS1 (NCBI acc. KX129910) and *Ga*CHS2 (NCBI acc. KX129911) with an ORF of 1176 and 1170 bp, respectively. *Ga*CHSs were heterologously expressed and purified in *E*. *coli* to validate their functionality. Functionality of CHS isoforms was also characterized via enzyme kinetic studies using five different substrates. We observed differential substrate specificities in terms of their K_m_ and V_max_ values. Accumulation of flavonoid constituents naringenin and quercetin were also quantified and their relative concentrations corroborated well with the expression levels of *Ga*CHSs. Further, our results demonstrate that *Ga*CHS isoforms show differential expression patterns at different reproductive phenological stages. Transcript levels of *Ga*CHS2 were more than its isoform *Ga*CHS1 at the anthesis stage of flower development pointing towards its probable role in male reproductive maturity.

## Introduction

Flavonoids are important natural products fashioned by evolution in plants in varied forms during their shift from water to land. These are utilized for multitude of purposes by the plants. Elevating figures of more than 9000 different flavonoids have been identified [1]. They are generally classified into major subgroups including chalcone, flavones, flavonols, flavandiols, anthocyanins, proantho-cyanidins or condensed tannins and the aurones [2]. Almost all leguminous and a few non-leguminous plants possess specialized flavonoids called isoflavonoids [3]. Flavonoids are of particular interest in plant reproduction as they perform main role in pigmentation and pollination. Flavonoids being important constituents of pollen and pistil play a significant role in the fertility and sexual reproduction [4]. Pollination experiments in tobacco have elucidated the role of flavonoids as necessary phytochemicals for pollen maturation, pollen tube growth, fertilization andseed development [5]. Further evidence for the role of flavonols in sexual reproduction has been provided by the male sterile petunia white anther (*wha*) mutant. This mutant showed complementation by the introduction of a functional CHS cDNA [6]. The reduced level of flavonoids in tomato led to lack of functional fertilization and resulted into seedless fruits [7]. The pollen coat has the direct contact to the biotic and abiotic environments. It contains flavonoids and flavonol glycosides required not only for fertilization but also for protection against biotic and abiotic stressors.

**Fig 1:**
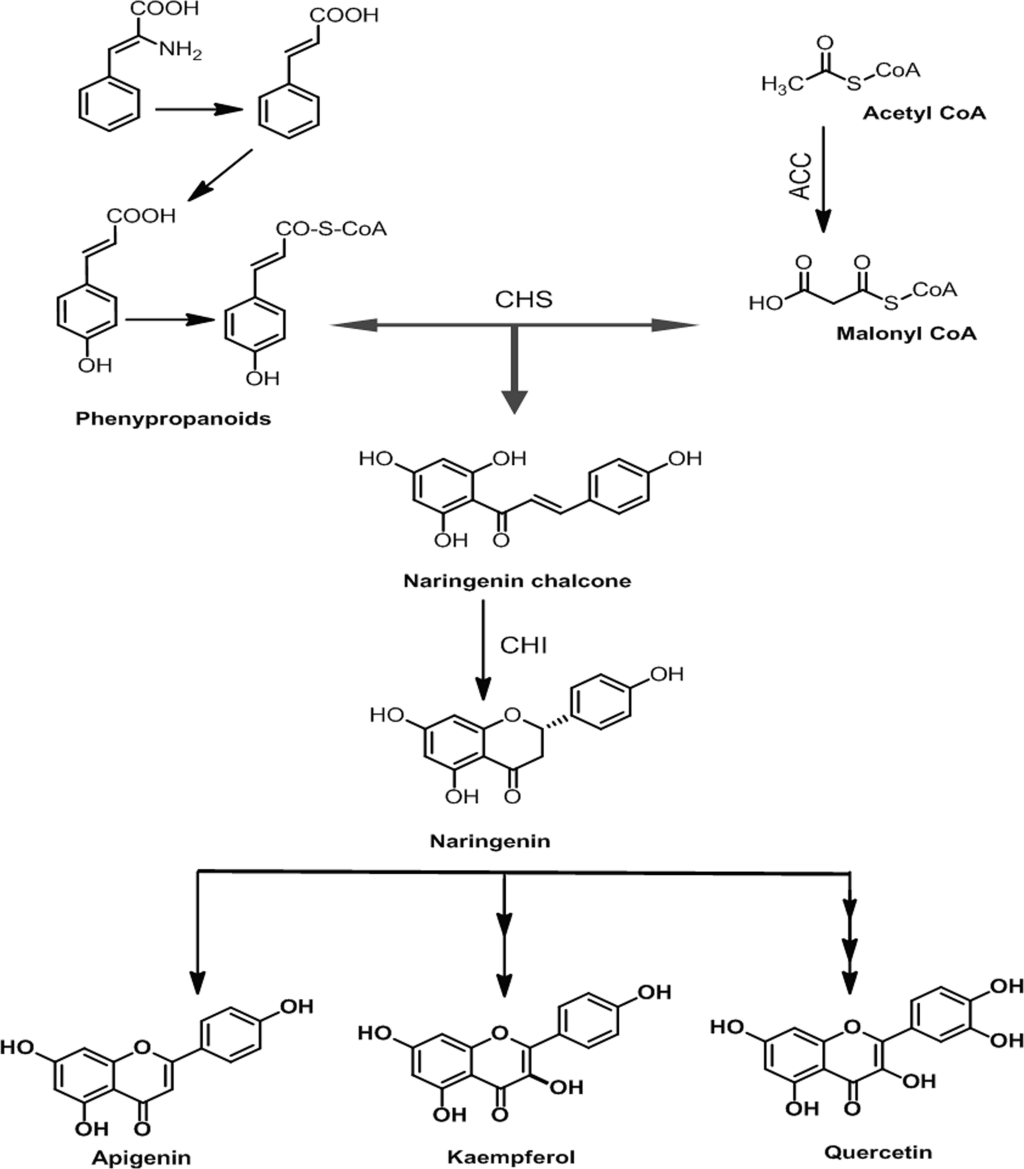
Flavonoid biosynthetic pathway. A schematic representation of precursors used in the biosynthesis of flavonoids, flavones and flavonols with some important enzymes involved in flavonoid biosynthetic pathway. Abbreviations: ACC = Acetyl-CoA carboxylase;PAL = phenylalanine ammonia lyase; CHS = chalcone synthase and CHI = chalcone isomerase.

Amongst the diverse structural and regulatory enzymes active in flavonoid biosynthetic pathway, chalcone synthase (CHS, EC 2.3.1.74) is the focal and thoroughly studied enzyme. CHS gene was first isolated from Parsely (*Petroselium hortense*) [8] which followed the identification and characterization of about 20 functionally different CHS superfamilies of bacterial and plant origin [9]. From both monocotyledon and eudicotyledon plant species, about 650 CHS and CHS-allied genes have been isolated and sequenced [10,11]. These genes have been well characterized in *Petunia* hybrid [12], *Phalaenopsis* Orchid [13] and *Lilium* hybrid [14]. The reaction mechanism of CHS includes an acyltransferase activity loading starter moiety (p-coumaroyl CoA) onto the active site catalytic residue Cys, a malonyl CoA activating decarboxylative activity, a repetitive condensing activity which joins the acetyl anion to growing ketide chain, a cyclase activity involving intramolecular Claisen condensation of the linear tetraketide intermediate to synthesize a cyclized polyketide precursor of chalcone, and finally ends with an aromatase like activity [15]. Moreover, in CHS mechanism, structural and functional evidence supports that p-coumaroyl CoA initiates the reaction instead of the extender unit malonyl CoA “Fig 1”.

Some extensively studied CHS genes include those from *Oryza sativa*, *Medicago sativa*, *Zea mays* and *Rehumemodi* [11,16]. These genes are predominantly expressed in flowers and at different developmental stages [12,17,18,19]. They are also expressed under different environmental conditions like Light [18,20,21,22], stress like wounding [23], and tissue specific [23,24,25] in a range of plant groups. Furthermore, diverse members of this family were observed to display unusual spatial and temporal expression patterns [10]. Studies have shown that CHS genes are differentially expressed in different plant tissues [26] and are also controlled by the plant circadian clock [27]. Amongst the three CHSs of *Gerbera hybrida*, two genes *G*CHS1 and *G*CHS3, exhibit exclusive expression through corolla development, while the third one *G*CHS2, showed more or less expression in all the tissues at different times [28].

*Grewia asiatica* L. (Malvaceae), commonly known as ‘Phalsa’ is a multipurpose gregarious shrub native to southern Asia, distributed from Pakistan east to Cambodia and widely cultivated in various tropical countries [29]. *G*. *asiatica* plant in general and fruit in particular is reputed for its medicinal properties as it finds mention in Ayurveda and is being used in various ailments in the Indian Systems of Medicine [30].

In spite of the diverse uses, two drawbacks prevent full exploitation of this species. These are short shelf life of its fruits and larger seed volume as compared to fruit pulp. Short shelf life makes the fruits suitable only for local marketing and the larger seeds reduce the fruit volume. Moreover, there are no recognized suitable cultivars available for *G*. *asiatica* [31]. To circumvent these drawbacks, induction of parthenocarpy or stenospermocarpy is a viable option for increasing the shelf life and pulp volume of fruits.

In this direction, present study reports cloning and characterization of two isoforms of chalcone synthase genes from *G*. *asiatica*. The investigation herein also reports the differences in structure and enzyme activity using the purified recombinant proteins from the heterologous system (*E*. *coli*). Also, functionality of these CHS genes was characterized via enzyme kinetic studies by using five different substrates. The accumulation pattern of flavonoids assayed through HPLC analysis was corroborated with the mRNA transcript levels of *Ga*CHSs. These results demonstrate the role of *Ga*CHSs in biosynthesis of flavonoids during different vegetative and reproductive phenophases in the life cycle of *G*. *asiatica*.

## Materials and methods

### Plant selection and RNA isolation

The material for present study comprised *G*. *asiatica* plantation raised from seeds in nursery beds at CSIR-Indian Institute of Integrative Medicine, Jammu, India (32°.44'N 75°.55'E; 305 m in altitude) where the annual temperature fluctuates between 5°C and 45°C and mean annual rainfall measures upto 1100 mm. Total RNA was isolated using modified CTAB method [32,33]. The RNA quality was assessed by electrophoresis on 1% formaldehyde agarose gel followed by determining the absorbance ratio (A_260/280_) using spectrophotometer (Astra Auriga, Cambridge, UK).

### Primer designing and cDNA synthesis for cloning of *Ga*CHS1 and *Ga*CHS2

For cDNA synthesis, 3 μg of DNase I treated total RNA was reverse transcribed using Revert-aid premium reverse transcription kit (Fermentas, Burlington, Canada) with a modified Adapter-oligo-(dT) primer. The reaction set in a total volume of 20 μl containing 3 μg of total RNA, 10 μM oligo(dT) primer, 1X first strand buffer (250 mM Tris-HCl, pH 8.3; 250 mMKCl; 20 mM MgCl_2_; 50 mM DTT), 10 mM dNTPs and 1 μl of Moloney murine leukemia virus reverse transcriptase (200 units/μl) was incubated for 60 min at 42°C followed by 5 min at 70°C to inactivate the reverse transcriptase.

Degenerate primers were designed based on highly conserved regions of amino acid sequences of other plant CHSs retrieved from the GenBank data base at NCBI (National Centre for Biotechnology Information) using Blastn/BlastX [34] and ClustalW2 [35] programmes “Table 1”. RT-PCR for core amplification was carried out by using cDNA as template, under the following cyclic conditions: 1 cycle of 95°C for 3 min; followed by 35 cycles of 95°C for 30 s, 56°C for 45 s and 72°C for 1min; and a final extension of 72°C for 10 min in a thermal cycler (Eppendorf AG, Hamburg, Germany). The selected amplicons were separately cloned into pTZ57R/T vector (Fermentas, Burlington, Canada) and transformed into an *Escherichia coli* host strain (DH5; Invitrogen, Merelbeke, Belgium). The screened amplicons were sequenced (ABI PRISM 3130XL genetic analyzer; Applied Biosystems, Foster City, CA, USA) and sequence analysis was performed to ensure homology by using Blastn [34] programme.

**Table 1:** Primers used in this study.

Name	Sequence	Application
**Degenerate primers**		
DegCHS1_F	5′- TCAATGATCAAGAACGTT	Core amplification
DegCHS1_R	5′- TCACGAAGGTGACCGTC	Core amplification
DegCHS2_F	5′- ATCAAAGAATGGGGACAG	Core amplification
DegCHS2_R	5′- GCGGTGATCTCCGAGCAA	Core amplification
5′ and 3′ RACE primers		
GeneRacer RNA Oligo	5′-CGACUGGAGCACGAGGACACUGACAUGGACUGAAGGAGUAGAAA	RACE programme
GeneRacer Oligo dT	5′-GCTGTCAACGATACGCTACGTAACGGCATGACAGTG(T)_24_	RACE programme
5′ RACE_OUT*	5′-CGACTGGAGCACGAGGACACTGA	RACE programme
5′ RACE_INN*	5′-GGACACTGACATGGACTGAAGGAGTA	RACE programme
3′ RACE_OUT*	5′-GCTGTCAACGATACGCTACGTAACG	RACE programme
3′ RACE_INN*	5′-CGCTACGTAACGGCATGACAGTG	RACE programme
5′ CHS1	5′- ATCATTGATTGTCACACATGCGCTTAAATT	RACE programme
3′ CHS1	5′- TCTCGCCAGGACTTCGCTGAGAACAA	RACE programme
5′ CHS2	5′- TAAGCGCACATGTTTGGATTTCCTT	RACE programme
3′ CHS2	5′- ACCCTCCGTCAAGAGGTTCATGATGTA	RACE programme
Full-lengthprimers		
FulCHS1_F	5′-ATGGCGCCACCGTGCAGGA	Full length cloning
FulCHS1_R	5′- TCAATTGGCGGTGGGAACACT	Full length cloning
FulCHS2_F	5′- ATGGTGACAGTGGAGGAAG	Full length cloning
FulCHS2_R	5′- TTAAGCAGAGATACTATGA	Full length cloning
Expression primers		
ExpCHS1_F #	5′-GGATCC ATGGCGCCACCGTGCAGGA	Expression analysis
ExpCHS1_R #	5′-GAATTC TCAATTGGCGGTGGGAACACT	Expression analysis
ExpCHS2_F #	5′-GGATCC ATGGTGACAGTGGAGGAAG	Expression analysis
ExpCHS2_R #	5′-GAATTC TTAAGCAGAGATACTATGA	Expression analysis
Real-Time primers		
Actin_F	5′-GAGAGTTTTGATGTCCCTGCCATG	Real-Time analysis
Actin_R	5′-CAACGTCGCATTTCATGATGGAGT	Real-Time analysis
β-Tubulin_F	5′-CTGCCATCTTCCGTGGAAAGG	Real-Time analysis
β-Tubulin_R	5′-GACGAAGTACGACGAGTTCTTG	Real-Time analysis
RtCHS1_F	5′- TAGTCTGCACCAGGCATGTCTA	Real-Time analysis
RtCHS1_R	5′- TAGTCGGCGCCGGGCAT	Real-Time analysis
RtCHS2_F	5′- TAGACATGCCTGGTGCAGACTA	Real-Time analysis
RtCHS2_R	5′- TAGTCTGCACCAGGCATGTCTA	Real-Time analysis

*Primers provided with the kit

# enzyme sites are underlined

### RACE and full-length cloning of *Ga*CHS1 and *Ga*CHS2

The sequenced core amplicons were later used for designing gene specific primers (GSPs) to perform 5′ and 3′ RACE using Gene Racer cDNA amplification kit according to the product manual (Invitrogen, USA). Each of the 5′ and 3′ cDNAs obtained were separately used to obtain the flanking regions of core amplicons in two sets of PCRs. The first reaction set was carried out using 5′/3′ RACE adapter primer (5′/3′ RACE_OUT) and 5′/3′ CHS1 and 5′/3′ CHS2 GSPs “Table 1”, while as in the second set the amplified products from first set were subjected to PCR using inner adapter primers (5′/3′ RACE_INN) and 5′/3′ CHS1 and 5′/3′ CHS2 GSPs “Table 1”. Both initial and nested PCR reactions were carried out in a 50 μl reaction volume containing 1 μl cDNA as template, 2.5 μl each of 10 μM adapter primers and GSPs for respective reactions and 44 μl of master mix (33.5 μl MQ water, 10 mM Tris HCl; pH 9.0, 50 mMKCl, 2.5 mM MgCl_2_, 0.2 μM dNTPs and 2.5 U of Taq DNA polymerase). Thermo-profile for both initial and nested PCR amplifications was as follows: 3 min at 94°C, 35 cycles (30 s at 94°C, 30 s at 60–65°C, 1 min at 72°C) and 10 min at 72°C. The 5′ and 3′ nested amplicons obtained after RACE strategy were sub-cloned into pTZ57R/T vector and further sequenced. All the sequences of core and 5′/3′ fragments were aligned and subsequently analyzed using Blastn/BlastX [34] tools to validate the anticipated target CHSs.

By comparing and aligning the sequences of the core fragments, 5′ RACE and 3′ RACE products, the full-length cDNAs of *Ga*CHS1 and *Ga*CHS2 were generated and subsequently amplified with full length primers *viz* FulCHS1_F/FulCHS1_R and FulCHS2_F/ FulCHS2_R “Table 1”. A high fidelity DNA polymerase (New England Biolabs, Herts, UK) was used for amplification of complete ORFs under the following thermocyclic conditions: 1 cycle for 3 min at 94°C; followed by 35 cycles of 94°C for 30 s, 55–58°C for 35 s, and 72°C for 1:30 min; and a final extension of 10 min at 72°C. The resulted amplified full length ORFs were ligated in pJET vector (Fermentas, Burlington, Canada) and subcloned into *E*. *coli* DH5α.

### Bioinformatic analysis

The *in silico* analysis was done by using different Bioinformatics tools. The complete cds were translated using Translate tool [36], secondary structures were envisaged by SOPMA [37] and the characteristics of the deduced amino acid sequences were estimated using ProtParam and Compute pI/Mw [38] tools. The identification of structurally and functionally important residues in protein sequences were predicted by using ConSeq and ConSurf servers [39]. The 3D protein structures were determined by Phyre^2^ server using the crystal structure of *Medicago sativa* CHS as template [40]. The 3DLigandSite server was used to determine ligand binding sites [41]. PyMOL a Molecular Graphics System, Version1.7.4 Schrödinger, LLC was used for building the three dimensional protein structures (http://www.pymol.org/). Moreover, the amino acid sequences were aligned using Clustal Omega Multiple Sequence Alignment tool [42] and the phylogenetic analysis was performed using the ClustalW [35] program and MEGA 6 software based on the maximum likelihood method with 500 bootstrap replicates to obtain confidence level with the branches [43].

### Heterologous expression of recombinant *Ga*CHSs

The *Ga*CHS1 and *Ga*CHS2 ORFs were tailored by adding BamHI and EcoRI restriction sites upstream to start and downstream to stop codons respectively using sense and antisense primers. The resulting CHSs were cloned with BamHI and EcoRI and excised from pJET vector (Fermentas, St. Leon-Rot, Germany). The clones were further confirmed by sequencing prior to their subcloning into the restriction sites of pre-digested and purified bacterial expression vector pGEX-4T-2. The cloned CHS proteins were expressed as fusion proteins with GST-tag at N-terminus of the expression vector. The heterologous expression of the recombinant proteins was carried out as described earlier [11].

### Enzyme purification

Protein expression was induced with 1.0 mM IPTG at 37°C by growing *E*. *coli* BL21 (DE3) cells transformed with respective expression plasmids of *Ga*CHS1 and *Ga*CHS2 in LB at A_600_ = 0.4–0.6. Cells were grown further for 6–8 h at 30°C and then harvested by centrifugation (6000 g at 4°C for 10 min; Eppendorf, Hamburg, Germany). The harvested pellet was resuspended in 1XPBS solution (140 mMNaCl, 2.7 mMKCl, 10 mM Na2HPO4, 10 mM KH2PO4, pH 7.3) followed by lysis (adding 20 mM DTT and (0.2 mg/ml) lysozyme) for 30 min.The culture was briefly sonicated (3X30 sec) using probe sonicator (Sartorius, Gottingen, Germany) and incubated on ice for 30 min with 1% (v/v) Triton X-100. Further, soluble and insoluble fractions were separated by centrifugation (12,000 g; 4°C; 15 min). The supernatant was incubated overnight with glutathione-sepharose beads (1 ml L21 of culture) (GE Healthcare, Little Chalfont, UK) at 20°C. The beads were washed five times with 10 bead volumes of 1XPBS. To remove the glutathione S-transferase (GST) moiety, thrombin (4 U/ml of beads) was added to the beads, and cleavage was allowed to proceed for 10–12 h at 24°C. The beads were pelleted (600 g at 4°C for 5 min), supernatant containing proteins were incubated overnight further with benzymedene beads to remove the thrombin. The purified protein samples were denatured and analysed on 10% SDS-PAGE and their concentration was directly measured on spectrophotometer.

### Enzyme kinetic studies under *in vitro* conditions

The *in vitro* enzyme kinetic studies by examining the formation of product of purified *Ga*CHSs were determined individually through LC-MS analysis. The reaction mixture contained purified enzyme (30 μg), starter-CoA 60 μM each and a common extender unit malonyl-CoA 150 μM in a 100 μl reaction with 0.1 M potassium phosphate (pH, 7.0), 1 mM EDTA and 10% glycerol under standard conditions. The 60 μM starter-CoA molecules include p-coumaroyl-CoA, Acetyl-CoA, Butyryl-CoA, Hexanoyl-CoA and Octanoyl CoA. The reactions were incubated for 1 h at 30°C and quenched by acidification (20 μl of 20% HCl). The soluble fraction was collected by further extraction with ethyl acetate (3 X 200 μl). The extracts were redissolved in methanol prior to dryness through evaporation. For the identification of reaction products of the purified *Ga*CHS1 and *Ga*CHS2 proteins, naringenin and naringenin chalcone were used as reference compounds. To confirm and quantify naringenin and naringenin chalcone produced in the reaction, the extracts were subjected to LC-MS analysis.

The steady-state kinetic constants were determined from initial velocity measurements where product formation was linear over the monitored time periods, using standard assay conditions with a fixed malonyl-CoA concentration (120 μM) and varied starter-CoA concentrations (10–250 μM). Using GraphPad Prism 6 software, the kinetic constants K_m_ and V_max_ were calculated with nonlinear regression analysis.

### Product identification using HPLC-ESI-MS/MS analysis

The stock solutions (1 mg/ml) of naringenin and naringenin chalcone were freshly prepared in methanol, filter sterilized with 0.25 μm membrane filters (Millipore, Bedford, USA) and stored at 4°C until further use. Standard working solutions were obtained by making appropriate dilutions of stock solutions for the preparation of six point calibration curve. The analyses were performed using an Agilent 1260 Infinity (Agilent, USA) HPLC system equipped with 1260VL infinity quaternary pumps, autosampler and a thermostat compartment. The samples were separated on a Purospher STAR RP-18e column (100 x 4.6mm; 5μm particle size). Mobile phases consisted of 0.1% (v/v) formic acid in water (eluent A) and acetonitrile with 0.1% (v/v) formic acid (eluent B). A gradient programme was used as follows: 0–10 min, 50–80% B; 10–15 min, 80% B; 15–17 min, 80–50% B; 17–20 min, 50% B. The flow rate was adjusted to 0.3 ml min^-1^ and column temperature was maintained at 30°C. Triple-quadrupole tandem mass spectrometry (MS/MS) was carried out on an Agilent 6410 tandem triple quadrupole mass spectrometer (TQD-MS) equipped with an ESI ion source operating in both positive and negative ion mode. ESI source was operated in positive ionization mode and the quantification was performed in MRM mode. The MS parameters optimized were: capillary voltage of 4.0 kV and gas temperature 300°C. Nitrogen was used as desolvation gas at the rate of 12 l/min and nebulizer pressure was maintained at 50 psi. Nitrogen was also used as the collision gas. All the data were collected in the centroid mode and acquired and further processed using Mass Hunter work station software (Agilent). Several LC parameters were optimized to obtain better separation and higher sensitivity with reduced analysis time. Better peak separation was observed when acetonitrile was used as the organic phase. In addition, different concentrations of formic acid in water (0.01 to 0.5%) were tested, and the best peak shape and higher resolution was observed in aqueous phase with 0.1% formic acid. The high quality separation was achieved with Purospher STAR RP-18e column (100 x 4.6mm; 5μm particle size). MS scan mode conditions were optimized using the reference compounds and higher sensitivity and clear mass spectra were observed in analyses conducted in the positive ion mode. In positive ion mode, quasi-molecular ions [M+H]^+^ of naringenin and naringenin chalcone were generated, whose product ions were high with good specificity. The optimized fragmentor voltage and collision energy for both naringenin and naringenin chalcone were 130V and 17 eV, respectively. Quantification was performed in MRM mode having the ion transitions for naringenin and naringenin chalcone as *m/z* 273/153 and *m/z* 273/147, respectively. The developed method showed 14.9 min retention time for naringenin and for naringenin chalcone it was 13.8 min. Compounds were identified by comparison of molecular ion, fragmented ions (MRM) and retention time with that of the standard compounds.

### Tissue-specific and reproductive phenophase-specific gene expression analysis

The tissue-specific as well as flowering phenophase-specific expression profiling was done by quantitative real time PCR analysis. Total RNA was isolated from different parts (leaf, stem and root) of the plant and from different reproductive phenological stages of flower including bud initiation, bud growth, pre-anthesis, anthesis, senescence and fruit initiation. Different floral parts at anthesis stage including, sepals, petals, stamens, carpel and ovary were also subjected to total RNA isolation. For each sample, DNase-treated RNA (3 μg) was reversely transcribed using iScript cDNA synthesis kit (BioRad, California, USA) according to manufacturer’s instructions. The SYBR based chemistry using SYBR Premix Ex Taq (Takara, Dalian, Liaoning, China) was applied in ABI Step one real time quantitative PCR system (Applied Biosystems, Foster City, CA, USA) to run the PCR reactions. The respective PCR reactions of 10 μl included 0.5 μl of cDNA as template, 0.2 μM each of the primers (Table 1), 5 μl of SYBR Premix Ex Taq and MQ water to make up the final volume. The reaction thermo-profile was followed as recommended by the manufacturer: holding stage of 1 cycle at 95°C for 10 min, cycling stage (40 cycles) of 95°C for 15 s and 60°C for 1 min, and finally melting curve stage of 95°C for 15 s, 60°C for 1 min and 95°C for 15 s. The primer designing was done by Primer Express version 3.0 (Applied Biosystems) and were further validated by a dissociation curve (observation of a single peak for each primer pair). All the samples were run as biological as well as technical replicates. Two housekeeping genes, β-Actin and tubilin, amplified with Actin_F and Actin_R and Tubilin_F and Tubilin_R primers, respectively “Table 1” were used as endogenous control to normalize the expression of the selected genes. Average ct (cycle threshold) values of the two reference genes were used to normalize the data. The amplification of the target genes was monitored every cycle by SYBR green fluorescence. The Real-Time amplification data were exported to Microsoft Excel and further analysed by the Livak method [44] and expressed as normalized relative expression level (2^−ΔΔCT^) of the respective genes in various samples.

### Flavonoid extraction and quantification by HPLC

The above mentioned plant samples were dried under gentle air stream (temperature 25 ± 2°C and relative humidity 65 ± 5%) and pulverized to fine powder using mortar and pestle. The powdered samples were serially extracted (3 X 100 ml) with DCM: MeOH in the ratio of 1:1 (v/v). The extractions were done at room temperature over a period of 72 h (24 X 3) and every time fresh solvents were used for the left out marc. The filtrates were combined, filtered through Whatman No. 1 paper filter and solvents removed at 45°C under reduced pressure using a rotary evaporator (Sigma Aldrich, USA) to yield the extract. The stock solutions (1 mg/ml) of naringenin and quercetin along with extracts were freshly dissolved in methanol and filter sterilized with 0.25 μm membrane filters (Millipore, Bedford, USA). The HPLC (Shimadzu CLASS-VP V 6.14 SPI model) equipped with RP-18e column (E-Merck, 5μm, 4.6 × 250 nm), a photo-diode array detector (SPD-M10A VP model) and a pump (LC-10AT VP model) was used for the analysis of flavonoid constituents. A standard method [45] with slight modifications was used for the determination of flavonoid constituents (naringenin and quercetin) in different plant samples. The solvent system was 97.8% (v/v H2O), 2% CH3CN, 0.2% H3PO4 (A) and 97.8% (v/v CH3CN), 2% H2O, 0.2% H3PO4 (B) with a gradient elution of 0–30 min, 20% B; 30–35 min, 45% B; 35–38 min, 55% B; 38–40 min, 55% B; and 40–45 min, 20% B; at a flow rate of 0.6 ml/min. Injection volume of the sample was 20 μl and the column temperature 30°C. The identification, detection and quantification of flavonoids were done on the basis of retention time of reference compounds under a specific set of column operating conditions. Elution positions were established with authentic samples and by comparison with literature data. Relative contents of different flavonoid constituents were determined and expressed as percentage peak area. The flavonoid content was monitored at 285 nm and 370 nm for naringenin and quercetin respectively.

### Statistical analysis

All the experiments were analyzed with at least three replicates. The values of flavonoid content and gene expression investigation were expressed as mean ± standard deviation (SD). Statistical analyses were carried out by one-way analysis of variance (ANOVA) and the statistical significance was considered at P < 0.001.

## Results

### Cloning of *Ga*CHSs

The amplified, cloned and sequenced core fragments were used for designing RACE primers followed by PCR strategy to obtain complete cds sequences of *Ga*CHS1 and *Ga*CHS2. The core fragments of 400 bp each were obtained using degenerate primers which were amplified on both sides using 5′ and 3′ RACE primers, and the full-length cDNA sequences were obtained by further amplification using full-length GSP’s (Table 1). The nucleic acid sequence alignment of the full-length CHS sequences revealed sequence similarity to related plant CHSs through Blastn/Blastx analysis tools. The isolated genes were designated as *Ga*CHS1 and *Ga*CHS2 with an ORF of 1176 and 1170 bps, respectively. The sequences were submitted to NCBI data base with accession numbersKX129910 and KX129911, respectively. The amino acid sequences of full-length cDNAs of *Ga*CHS1 and *Ga*CHS2 were shown to display sequence similarity (40–70%) with orthologous sequences of chalcone synthase from different plant species including *Hibiscus cannabinus* (GenBank: AIA22214.1), *Theobroma cacao* (GenBank: EOY09158.1), *Gossypium raimondii* (GenBank: XP_012436331.1), *Gossypium arboreum* (GenBank: KHG05952.1) and *Abelmoschus esculentus* (GenBank: AGW22222.1) using Blastx/Blastp algorithm.

### Characterization of *Ga*CHSs through *in silico* analysis

The ORFs of *Ga*CHS1 (1176) and *Ga*CHS2 (1170) were subjected to translate tool to generate the primary amino acid sequences of 391 and 389 amino acids respectively each corresponding to a protein of about 43kDa with a calculated pI of 10.14 and 7.62 respectively. The first AUG coding for methionine was deliberated as the initiation codon as per the rule. Using Clustal Omega web tool, the primary structures of the two CHS isoforms were deduced. The pair wise alignment of the two showed 73% identity at nucleotide and 62% at the amino acid level respectively. The secondary structure analysis by SOPMA revealed that *Ga*CHS1 and *Ga*CHS2 showed different patterns with a respective percentage for α-helices: 25.06%, 42.16%; random-coils: 57.29%, 30.08%; beta turns: 6.65%, 10.80%; and extended strands: 11.00%, 16.97%. The signal peptides as supported by SignalP 4.1 and TMHMM servers were absent in both the *Ga*CHSisoforms. Analysis of various functional residues of amino acid sequences by ConSurf program showed evolutionary conservation in *Ga*CHS1 and *Ga*CHS2 and the structural residues of the proteins were determined by ConSeq server “S1 Fig”.

**Fig 2:**
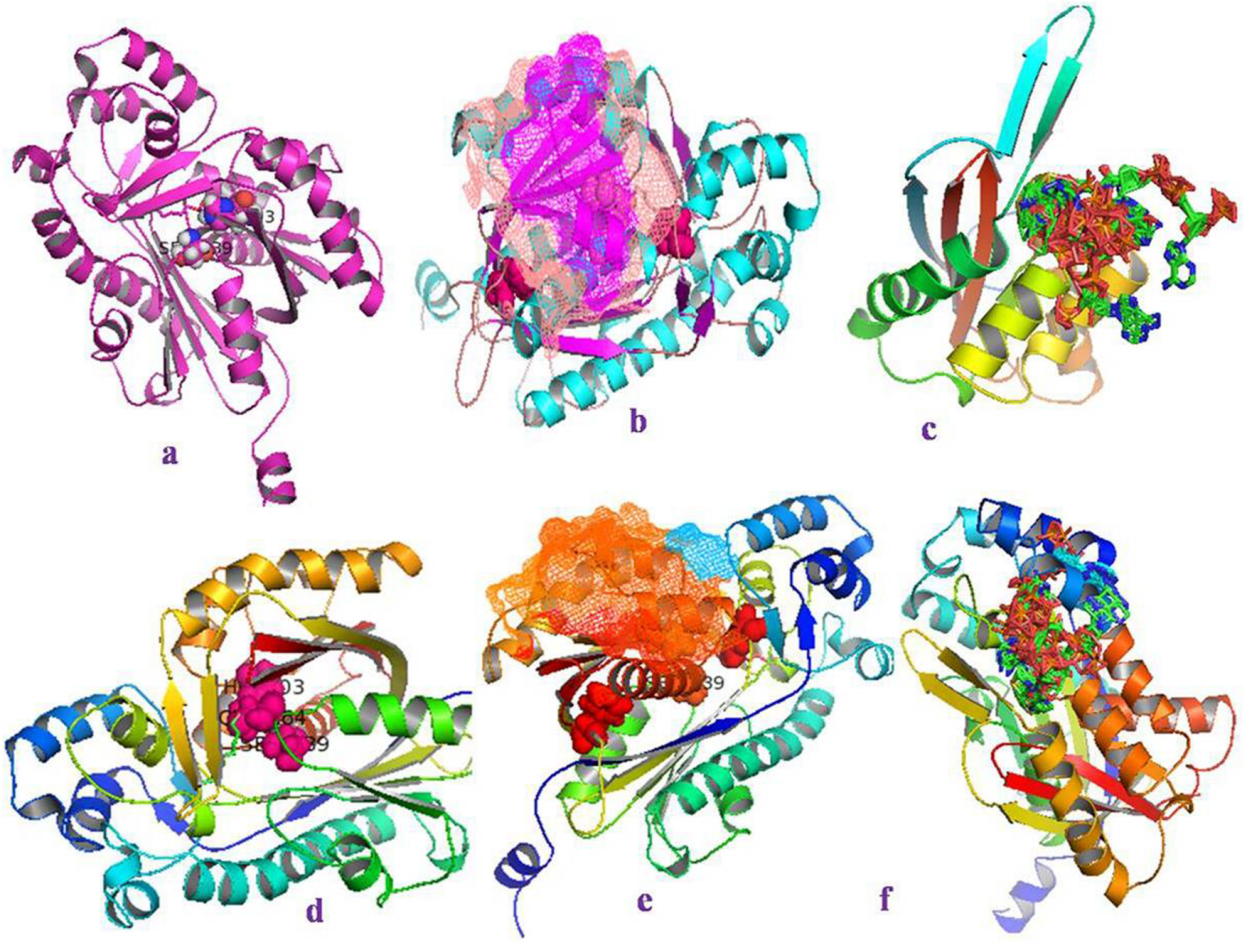
Predicted three-dimensional models and ligand-binding sites of *Ga*CHSs. Ribbon model display of the three-dimensional structures of *Ga*CHS1 and *Ga*CHS2 with conserved catalytic triad (Cys-His-Asn) shown in the central core of the structures **(a and d)**;Ribbon model display of the three-dimensional structures of *Ga*CHS1 **(b)** and *Ga*CHS2 **(e)** as predicted by I-TASSER web server, showing geometry of active site a malonyl-CoA binding motif shown as mesh structures and gatekeepers Phe^215^and Phe^265^ (Pink in *Ga*CHS1 and Red in *Ga*CHS2). The ligand binding sites as predicted by 3DLigandSite web server are depicted in the ribbon model **(c and f)**. The predicted ligand binding sites for *Ga*CHS1 are Ala^211^, Gln^212^, Ala^213^, Leu^214^, Phe^215^, Ile^254^, Phe^265^, Leu^267^, Lys^269^, Val^271^, Pro^272^, Gly^305^, Gly^306^, Ala^308^, Ile^309^, Ile^336^ and for *Ga*CHS2, the predicted ligand binding sites were Lys^55^, Phe^56^, Asp^57^, Leu^58^, Ser^59^, Ala^60^, Val^62^, Thr^63^, Ile^64^, Leu^164^, Leu^206^, Asp^207^, Leu^209^, Val^210^, Gly^211^, Leu^214^, Phe^215^, Ile^254^, Phe^265^, Leu^267^, Lys^269^, Val^271^, Pro^272^, Gly^305^, Gly^306^, Ala^308^, Asn^336^.

**Fig 3:**
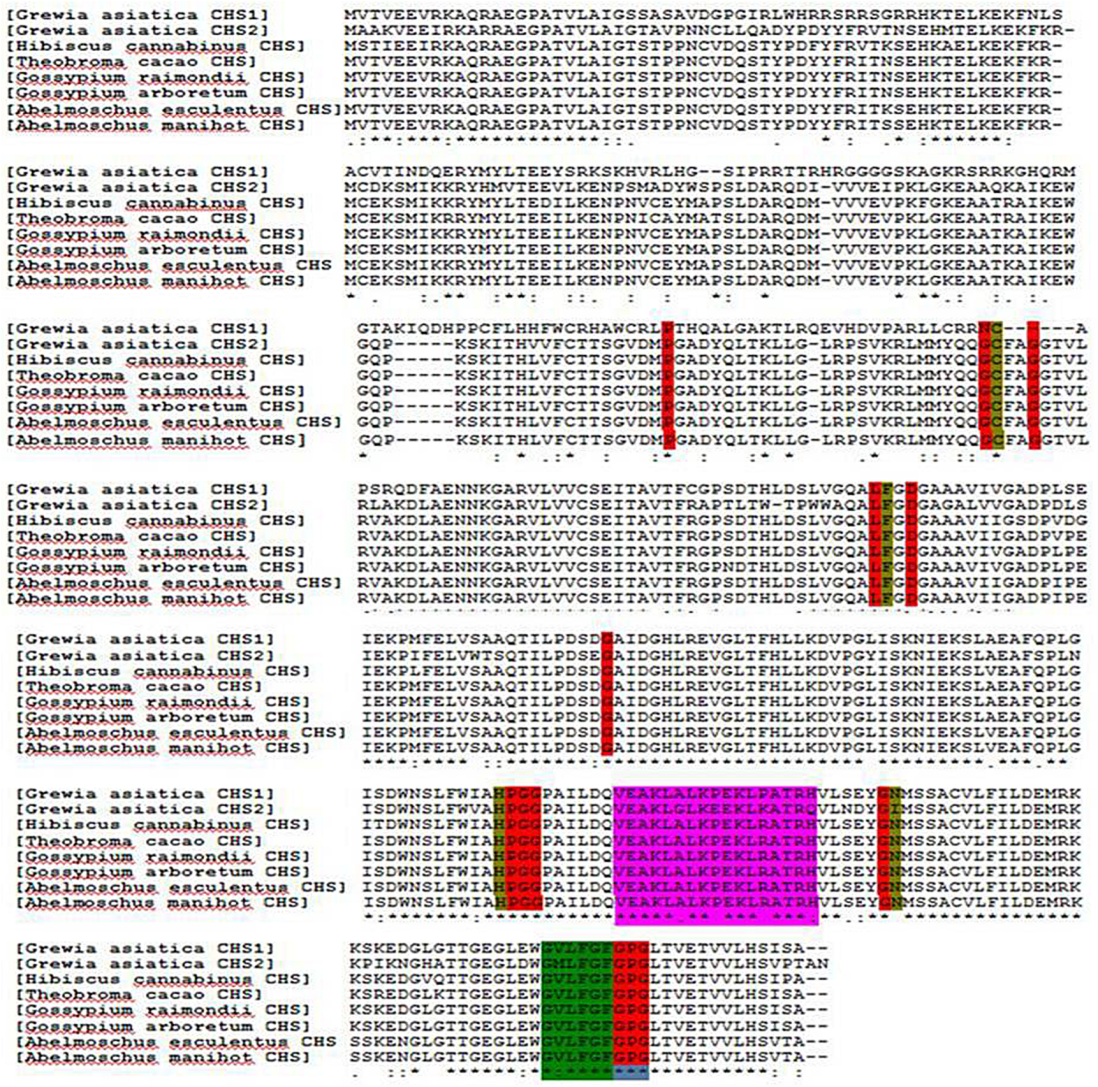
Multiple sequence alignment of deduced amino acid sequencesof *Ga*CHS-1 and *Ga*CHS-2 with related plant CHS sequences using Clustal Omega multiple sequence alignment tool. Functionally important conserved residues are highlighted with a coloured background: Dark yellow, the four catalytic residues (Cys-His-Asp triad + F) that are conserved in all chalcone synthases; red, the 13 residues that shape the geometry of the active site; pink, the malonyl-CoA binding motif; and green, the highly conserved CHS signature sequence, N-myristoylation motif. (GenBank accession numbers): *Grewiaasiatica*CHS-1(KX129910), *Grewiaasiatica*CHS-2 (KX129911),*Hibiscus cannabinus*CHS1 (AIC75908.1), *Bv*CHS, *Theobroma cacao* (EOY05368.1), *Gossypiumraimondii*(XP_012454899.1), *Gossypiumarboreum*(KHG14899.1), *Abelmoschusesculentus*(AGW22222.1), *Abelmoschusmanihot*(ACE60221.1).

The three dimensional structural models of *Ga*CHS1 and *Ga*CHS2 were determined by I-TASSER and Phyre2 web servers using the crystal structure of *Medicago sativa* CHS (PDB code c1cmla) as template. The I-TASSER based model of *Ga*CHS1 showed a confidence score (C-score) of 1.18, 0.57±0.15 TM-score (estimated accuracy of model) and a root mean square deviation (RMSD) of 9.5±4.6Å. Similarly, the C-score, TM-score and RMSD for *Ga*CHS2 were 1.40, 0.91±0.06 and 3.9±2.6 Å, respectively. PyMOL, was used to create and design all the 3D protein structures “Fig 2”. The 3D LigandSite tool was used to predict the amino acids constituting the ligand binding site with 16 and 27 residues identified in *Ga*CHS1 and *Ga*CHS2, respectively “Fig 2B and 2E”. Multiple sequence alignment revealed that the *Ga*CHSs maintain identical conserved catalytic triad Cys-164, His-303, and Asn-336 (marked with Dark yellow background) and a highly conserved Phe residues acting as gatekeepers in all the chalcone synthases, Phe^215^and Phe^265^(shown in 3D structure with a pink background in *Ga*CHS1 and red in *Ga*CHS2). In addition, *Ga*CHSs also contain 13 inert active site residues (marked with red background) that shape the geometry of active site, a malonyl-CoA binding motif (marked with pink background) and a highly conserved signature sequence GVLFGF (marked with green background) “Fig 3”.

### Phylogenetic analyses

**Fig 4:**
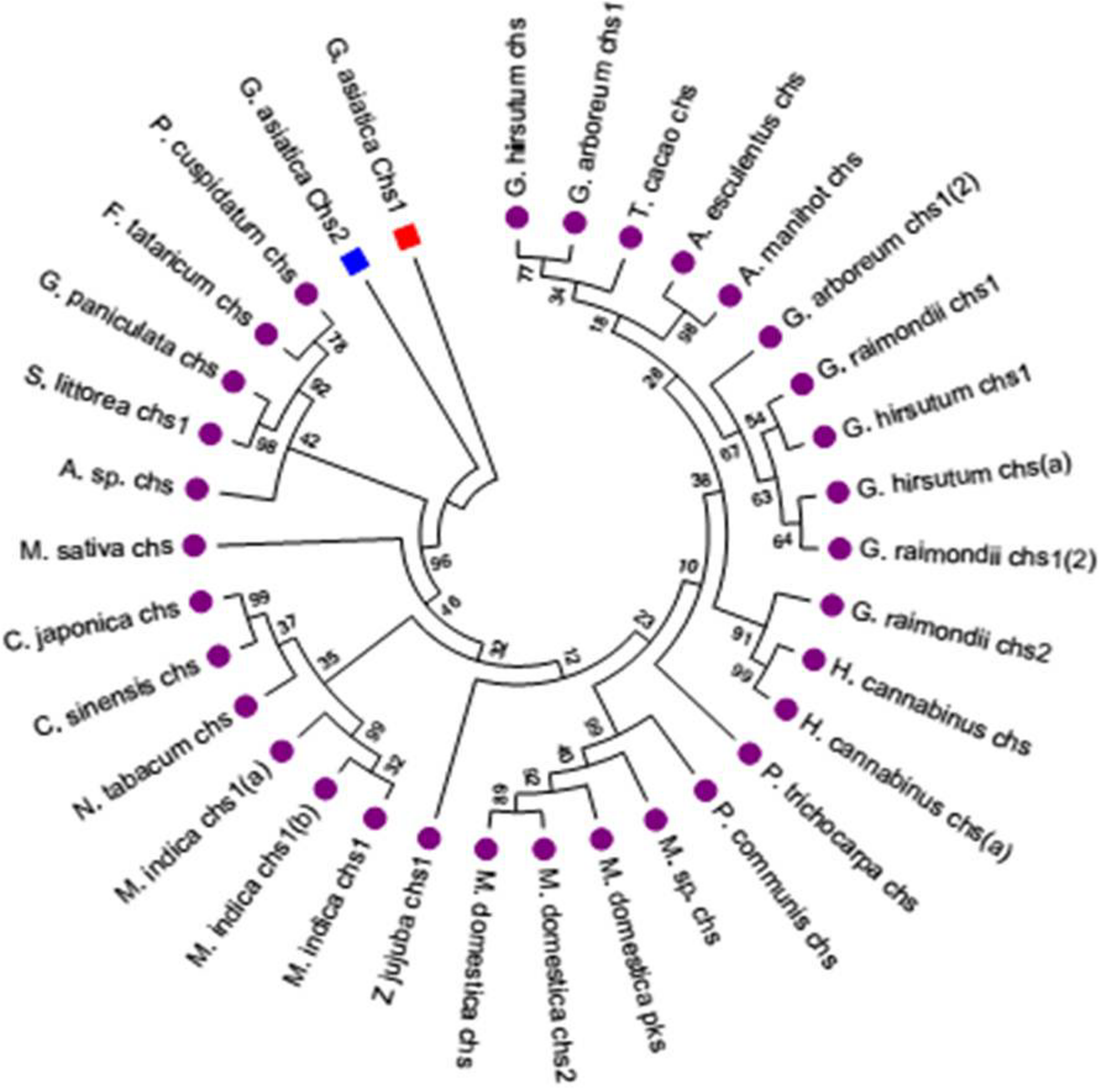
Phylogenetic analysis of *Ga*CHS-1 and *Ga*CHS-2. The phylogenetic analysis was performed based on Maximum Likelihood methodwith 1000 bootstrap replicates using the MUSCLE program and MEGA- 5 software. The analysis involved alignment of 34 amino acid sequences which were chosen by BLAST search of *Ga*CHS genes from NCBI data-base. The desired sequences were selected based on the complete cds information available. The evolutionary distances were computed using the Poisson correction method. The database accession numbers of the CHS sequences used are as follows: *Grewiaasiatica*CHS-1(KX129910), *Grewia asiatica*CHS-2 (KX129911), *Gossypiumhirsutum*(AEO96985.1), *Gossypiumarboreum* CHS-1 (KHG25969.1), *Gossypiumraimondii*(XP_012454899.1), *Theobroma cacao* (EOY05368.1), *Abelmoschusesculentus*(AGW22222.1), *Abelmoschusmanihot*(ACE60221.1), *Gossypiumhirsutum CHS2* (AEO96988.1), *Gossypiumraimondii*CHS1 (XP_012455000.1), *Gossypiumarboreum*(KHG14899.1), *Gossypiumhirsutum*CHS1 (ACV72638.1), *Gossypiumraimondii*CHS2 (XP_012440802.1), *Hibiscus cannabinus*CHS1 (AIC75908.1), *Hibiscus cannabinus*CHS2 (AIA22214.1), *Mangiferaindica*CHS1 (AIY24986.1), *Mangiferaindica*CHS (AIB06736.1), *Mangiferaindica*CHS (AIY24987.1), *Camellia sinensis*(AGI02994.1), *Camellia japonica* (BAI66465.1), *Ziziphus jujube* (XP_015887549.1), *Populustrichocarpa*(EEE78799.1), *Pyruscommunis* (AAX16494.1), *Malus hybridcultivar*(ACN25139.1), *Malus domestica*CHS2 (AFX71920.1), *Malus domestica*CHS1 (AGE84303.1), *Malus domestica*PREDICTED:polyketidesynthase5-like (XP_008380608.1), *Medicago sativa* CHS(AAB41559.1), *Silenelittorea* CHS1 (AMQ23617.1), *Nicotiana tabacum* CHS (AAK49457.1), *Gypsophila paniculata*CHS(AAP74755.1), *Polygonum cuspidatum* CHS (AFD64563.1), *Fagopyrumtataricum*CHS(ADG02377.1), *Arabidopsis*CHS(AAB35812.1). The bar indicates an evolutionary distance of 0.02%.

The phylogenetic relationship of *Ga*CHSs among themselves and with other orthologus CHS members was determined in order to get insights of evolutionary distance, a phylogenetic analysis of deduced primary amino acid sequences of *Ga*CHSs with related CHS proteins was performed with MEGA6 software based on neighbour joining method involving 1000 bootstrap replicates. About 33 amino acid sequences of CHS from both model and non-model plants were selected from different species submitted to NCBI data-base. The selected sequences ascertain the evolutionary history based on the complete cds information available (GenBankTM). Pairwise alignment of deduced primary structures of *Ga*CHS1 and *Ga*CHS2 showed that these are highly similar, with 62% identity at amino acid level and 73% identity at the nucleotide level. The CHS sequences of different species clustered with the homologous CHSs of the same species. Also, the two isoforms of *Ga*CHSs clustered with one another to form a single clade due to high similarity “Fig 4”.

### Expression analysis and purification of recombinant *Ga*CHSs

The biochemical characterization of *Ga*CHS gene products was performed by their sub-cloning into an IPTG inducible *E*. *coli* expression vector, pGEX-4T-2 under the control of Ptac hybrid-promoter. The expression level of proteins at different IPTG concentrations and different harvesting time intervals was checked on 10% SDS-PAGE. The highest expression level for each of the generated constructs was observed at 1.0 mM IPTG induction for 8 h at 30°C “S2 Fig”. The recombinant enzymes exhibited molecular mass of ~ 69 kDa which was related to the envisaged mass of recombinant fusion-proteins associated with GST (25.99 kDa). The optimum expression level (1.0 mM IPTG for 8 h at 30°C) was selected for further purification of respective proteins using the principle of affinity chromatography. The respective *Ga*CHS fusion-proteins (~ 69 kDa) were observed at corresponding ladder size on 10% SDS-PAGE “S3 Fig”. The fusion-proteins were reduced to their normal homo-dimeric size (~ 42 kDa) by cleavage at thrombin site located towards c-terminal of the GST tag.

### Enzyme kinetics and functional validation of *Ga*CHSs

**Fig 5:**
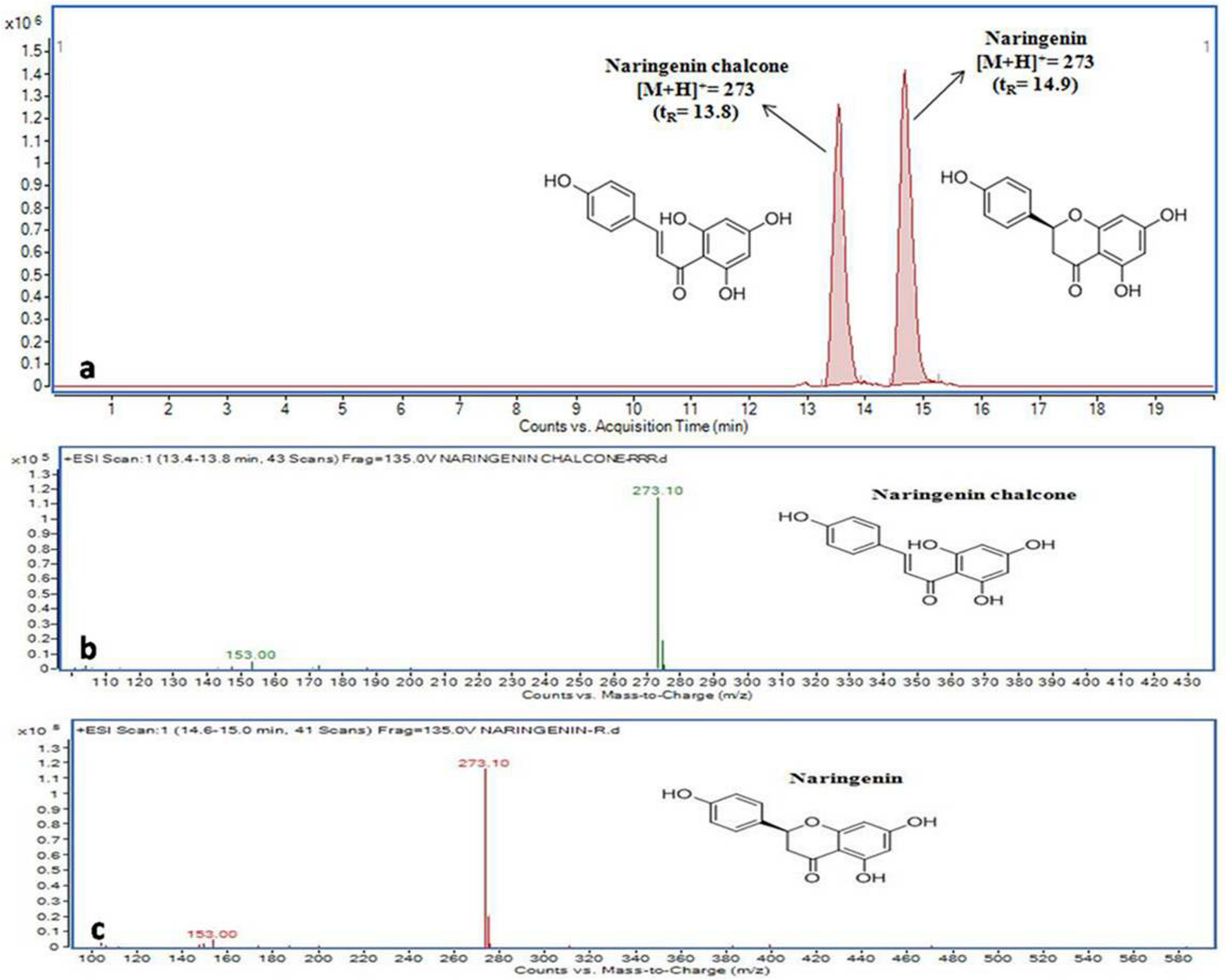
Multiple reactions monitoring (MRM) graphs. MRM chromatograms of standard compounds naringenin and naringenin chalcone eluting at 14.9 and 13.8 min, respectively; **(a),** MS spectra of naringenin chalcone **(b)** and naringenin **(c)**.

The known concentrations of purified *Ga*CHSs, were tested with extender substrate molecule malonyl-CoA and different starter-CoA substrate molecules namely p-coumaroyl-CoA, acetyl-CoA, butyryl-CoA, hexanoyl-CoA and octanoyl CoA for investigating the kinetic properties of the enzymes. The substrates and the reaction products were analysed by LC-MS in comparison to reliable standards of naringenin and naringenin chalcone. The enzymatic reactions of *Ga*CHSs resulted in the production of naringenin and naringenin chalcone with an expected retention time of 14.9 and 13.8 respectively using p-coumaroyl-CoA and malonyl-CoA. This is in conformity with the isolated cDNAs encoding CHS with representative enzymatic function “Fig 5A–5C”. The positive electron spray ionization (ESI)-ion mass spectrum resolved a molecular ion [M-H]^+^ at *m/z* of 273, similar to that of the reference compounds as depicted in the MRM graphs generated “Fig 5”. Furthermore, the outline of the fragmented form of [M-H]^+^ with the MRM conversion masses of *m/z* 273/153 and *m/z* 273/147 was roughly equal to that of the standards “Fig 5C”.

**Fig 6:**
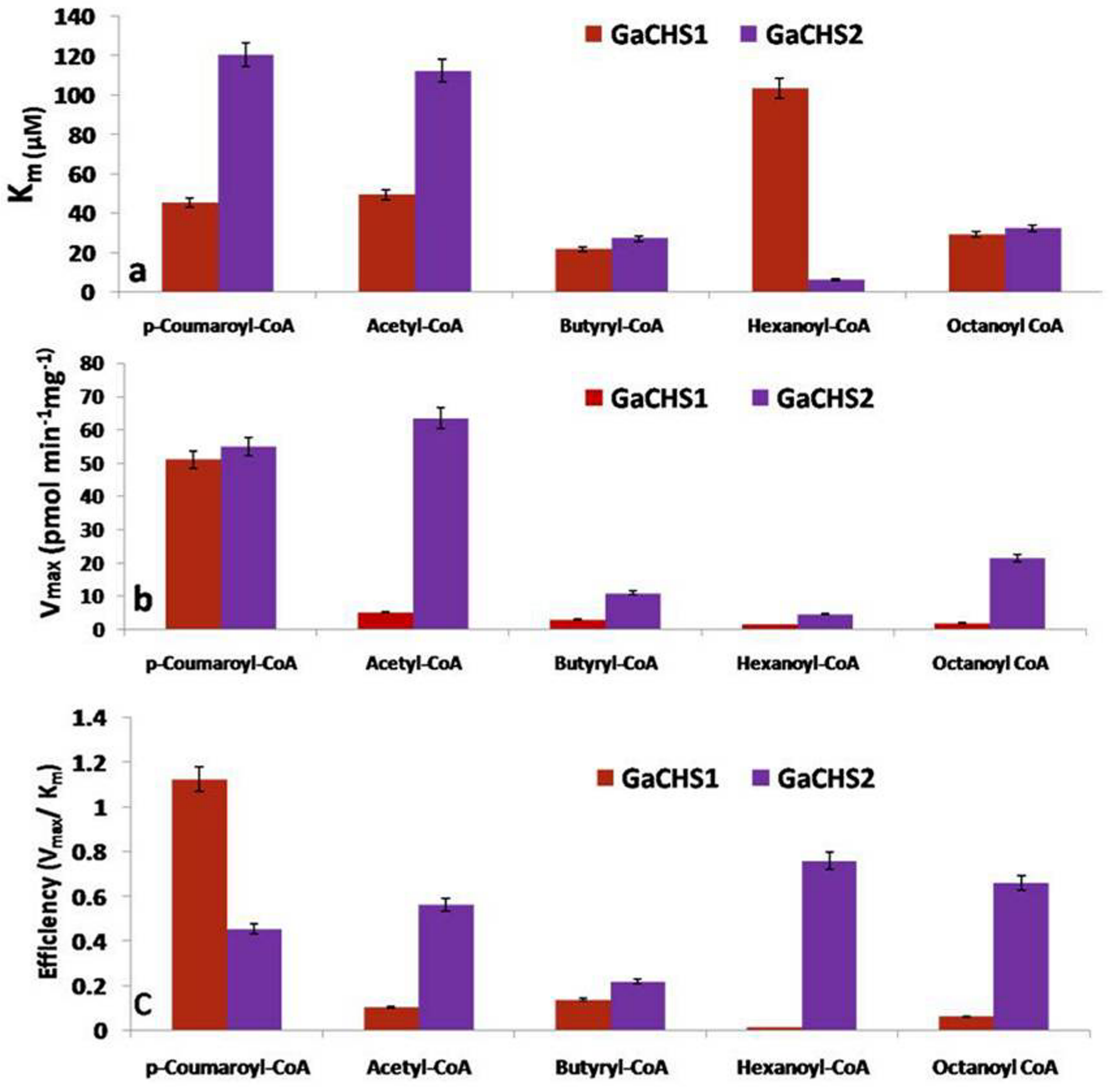
Kinetic study of *Ga*CHSs using different substrates. The kinetic parameters K_m_ and V_max_ were calculated by nonlinear regression analysis in order to determine the relative efficiency of *Ga*CHS1 and *Ga*CHS2 against the different substrates including p-coumaroyl-CoA, Acetyl-CoA, Butyryl-CoA, Hexanoyl-CoA and Octanoyl CoA.

With the aim of determining the steady-state kinetic parameters, standard assay conditions were used; purified protein (30 μg), the concentration of extender-CoA (120 μM) were kept constant by varying concentrations of starter-CoAs (10–250 μM). With the increase in substrate concentration there was a constant increase in product formation till the saturation limit of active site residues reached. The V_max_ values calculated with different starter-CoA substrates as calculated through non-linear regression analysis were different for *Ga*CHS1 and *Ga*CHS2. The evident K_m_ and efficiency (V_max_/ K_m_) values were different for *Ga*CHS1 and *Ga*CHS2 S4A & S4B Fig. *Ga*CHS1 displayed higher enzyme efficiency towards p-coumaroyl CoA as compared to other substrates which exhibited substantial efficiency with *Ga*CHS2. In general, K_m_ values of *Ga*CHS2 were higher as compared to that of *Ga*CHS1. Moreover, V_max_ values of *Ga*CHS2 were substantially many folder higher compared to that of *Ga*CHS1 “Fig 6”.

### Quantitative RT-PCR expression pattern of *Ga*CHSs

**Fig 7:**
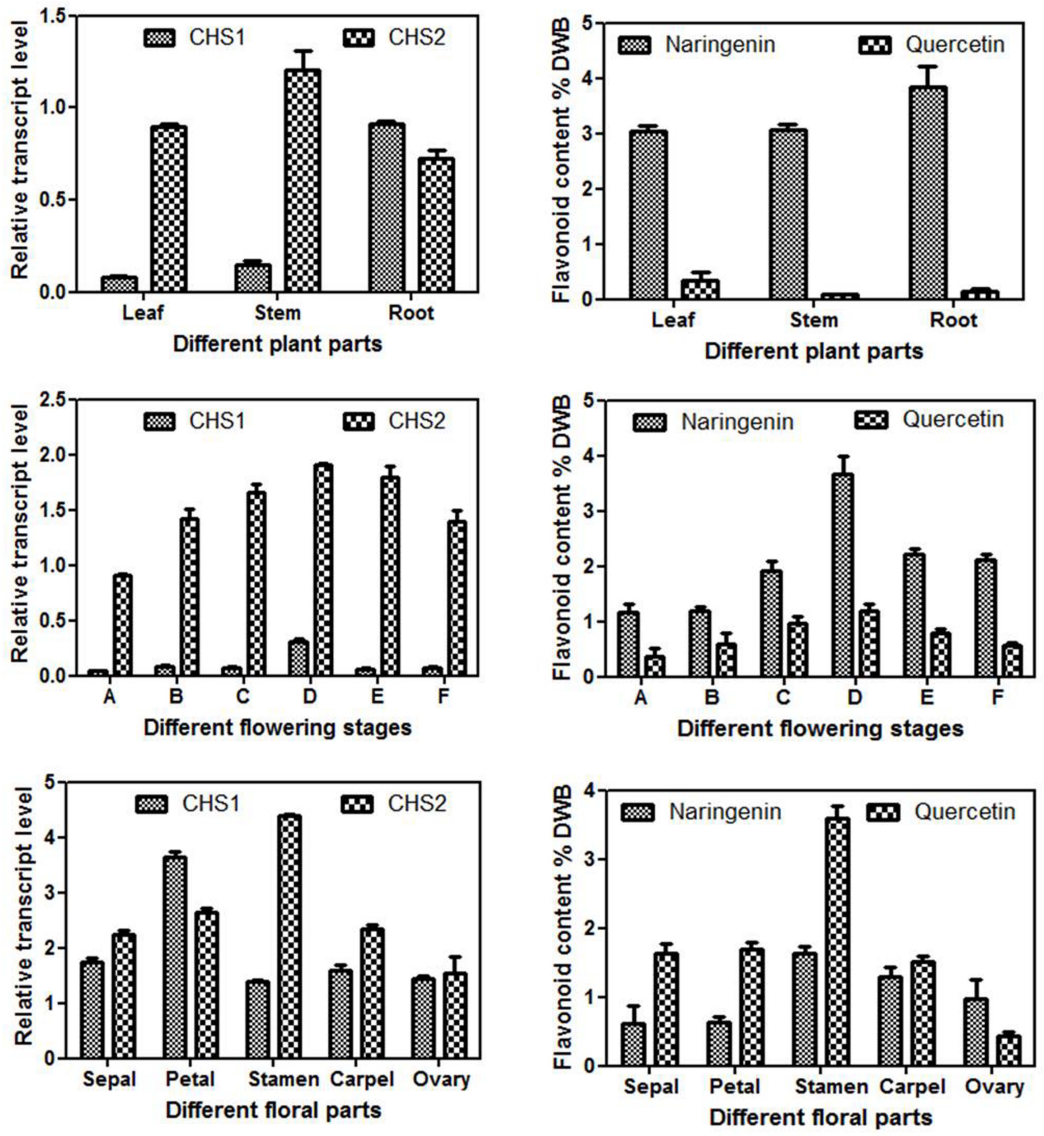
Real-Time expression analysis of *Ga*CHS1 and *Ga*CHS2and estimation of total flavonoid content in *Grewiaasiatica*. Quantitative estimation of the relative expression of *Ga*CHS1 and *Ga*CHS2 in leaf, stem and root tissues of *G*.*asiatica***(a).** Relative accumulation of flavonoids in leaf, stem and root tissues of *G*.*asiatica***(b).** Quantitative estimation of the relative expression of *Ga*CHS1 and *Ga*CHS2 in different floral phenological stages represented by (A) bud initiation, (B) bud growth, (C) pre-anthesis, (D) anthesis, (E) senescence and (F) fruit initiation **(c)** Corresponding relative accumulation of flavonoids in above mentioned floral phenological stages **(d)** Quantitative estimation of the relative expression of *Ga*CHS1 and *Ga*CHS2 in different floral tissues including sepals, petals, stamens, carpel and ovary **(e)** Relative accumulation of flavonoids in different floral tissues including sepals, petals, stamens, carpel and ovary **(f)**. The data were compared and analyzed with one-way analysis of variance (ANOVA) using GraphPad Prism 6 software. Values are expressed as mean ± standard deviation, with standard errors indicated by bars representing at least three replicates. The statistical significance was considered at P < 0.001.

The expression pattern of *Ga*CHS1 and *Ga*CHS2 in different tissues was examined using relative quantitative real time PCR (qRT-PCR) in order to understand the spatial regulation of the *Ga*CHS genes in *G*. *asiatica*. Although, the gene transcripts of two isoforms were expressed in each organ of *G*. *asiatica* and displayed a distinct expression pattern. The *Ga*CHS1 transcript levels were higher in root, followed by stem and leaf, whereas *Ga*CHS2 transcripts were more evident in leaves and stem than root “Fig 7A”. Among the different reproductive phenological stages of flower development, the transcript levels of *Ga*CHS2 were predominantly higher than *Ga*CHS1 at all the stages. *Ga*CHS1 expression was highest at post-anthesis stage of flower development while as, *Ga*CHS2 expression increased towards anthesis stage and remained almost invariable up to fruit set and then started declining “Fig 7C”. Both of the *Ga*CHS isoforms showed expression in different floral parts at anthesis stage of floral development. The expression of *Ga*CHS2 was observed in all the floral parts and the highest expression was in male part, stamens. *Ga*CHS1 transcript level was highest in petals “Fig 7E”.

### Determination of flavonoid constituents

To analyze the flavonoid composition of *G*. *asiatica*, methanolic extracts of different vegetative and reproductive plant tissue samples were subjected to HPLC analysis. Significant differences in the flavonoid composition of all the evaluated samples were observed. Naringenin and quercetin were found to be present in all the analysed samples. In vegetative tissues, naringenin content was higher than quercetin and among all the vegetative parts, it was found to be highest in roots (3.84±0.37 mg/g DWB, Dry Weight Basis) followed by stem (3.06±0.10 mg/g DWB) and leaf (3.03±0.10mg/g DWB). Leaves showed the maximum accumulation of quercetin (0.35±0.14 mg/gDWB) followed by root (0.14±0.05 mg/g DWB) and stem (0.086±0.015mg/g DWB) “Fig 7B”. In different reproductive phenological stages, both naringenin and quercetin contents were highest at anthesis stage (3.66±0.32 mg/gDWB) and (1.18±0.13 mg/g DWB) respectively which started declining towards fruit set “Fig 7D”. Among the different floral parts, the highest flavonoid accumulation was observed in male part of the flower (stamen) with naringenin and quercetin content as (3.78±0.39 mg/gDWB) and (1.80±0.16 mg/g DWB) respectively “Fig 7F”. Interestingly, in all floral tissues, quercetin content was highest in comparison to naringenin content.

## Discussion

The chalcone synthase, a homodimeric protein is structurally and mechanistically the simplest type III plant polyketide synthase, leading to the formation of an aromatic tetraketide, naringenin chalcone. The homology modelling, a predictive investigation, and sequence analysis studies of the *Ga*CHS isoforms revealed their similarity features with well-known characterized chalcone synthases signifying their generic CHS role. As per the extensive literature survey, CHS has been found to be present in all gymnosperms and angiosperms. The spatial and temporal distribution and specificity of downstream tailoring enzymes varies significantly across different species during the life cycle of an individual plant.

The well known conservation of CHS sequences across species was employed to recognize the catalytically important residues in *Ga*CHSs using Clustal Omega tool. The conserved amino acid residues present in almost all chalcone synthases were also found to be preserved in the primary amino acid sequences of *Ga*CHSs. Multiple sequence alignment analysis is employed for the determination of evolutionary divergence among genes through events like mutations, insertions, deletions and rearrangements under certain conditions. The conserved catalytic triad, previously characterized by other researchers from CHS, is important in shaping active site geometry and substrate selectivity [46]. The Cys-164 acts as a nucleophile in polyketide formation, whereas His and Asn carry out malonyl-CoA decarboxylation. The highly conserved Phe-215 residue plays an important part in substrate orientation at the active site [47].

The phylogenetic analysis of *Ga*CHS isoforms was evaluated with related CHS sequences from the different plant species belonging to family Malvaceae, Rosaceae, Salicaceae, Rhamnaceae, Theaceae, Anacardiaceae etc. These members were chosen owing to their maximum BLAST score with *Ga*CHS isoforms. The two isoforms *Ga*CHS1 and *Ga*CHS2 aligned with each other in a single clade and with the orthologus CHS members of the other plant species. The two isoforms differ in number of amino acids, *Ga*CHS1 with391 amino acids and the *Ga*CHS2 with389 amino acids. This indicates that the two isoforms may have evolved through gene duplication events. It has been observed in many plant species that the new CHS genes are evolved occasionally through gene duplication events [48]. The gene families of the species in phylogenetic investigation are subject to diverse evolutionary endings. Gene duplication event, a widespread feature of plant genomes is measured to be key mechanism for evolutionary advances and functional divergence [49,50]. Incidentally, gene duplication occurrence may be analysed as an essential resource for the origin of evolutionary advances [51]. There is growing evidence that the CHS-like enzymes in the CHS-superfamily evolved from CHS via duplication and subsequent divergence over the course of evolution [52,53]. The CHS and CHS-like genes provide an interesting example of evolutionary asymmetry and evolutionary novelty following gene duplication [54]. The different gene lengths of *Ga*CHSs, as a possible event of gene duplication may lead to evolutionary asymmetry between the two isoforms. The relationship between the degree of amino acid sequence identity and substrate specificity of the plant CHSs is highly complex and thus the phylogenetic relationships are not always an indicative measure of the possible role of these enzymes. It has been observed that diverse CHSs often share different substrates.

Previous studies have revealed multi-substrate catalyzing properties of CHS resulting in the formation of product as naringenin chalcone or naringenin by polymerizing various acyl-CoAs as starter and malonyl CoA as extender units [11]. In present study, the catalytic activity of purified *Ga*CHS isoforms was monitored by using different starter acyl-CoAs and extender malonyl CoA which resulted in efficient formation of the two isomers naringenin chalcone or naringenin. In all the cases, the product, naringenin chalcone was observed by using different substrates as detected through the MRM-LC-MS profile of the reaction samples. Retention times were effectively same to that of their authentic standards “Fig 5”. The *Ga*CHS2 was highly efficient with other substrates as compared to *Ga*CHS1 which showed maximum activity with starter p-coumaroyl-CoA and extender malonyl CoA. It has been observed that CHS shows wide substrate specificity range as it could accept both aliphatic and aromatic CoA esters to produce various reaction products including the unnatural aromatic polyketide [55]. The differential enzyme activity of different CHS isoforms with different substrates has also been reported in *Humulus lupulus* [56], *Gerbera hybrida* [57], *Emblica officinalis* [58] and others. The differential enzyme activity of *Ga*CHS2 towards different substrates indicates its evolutionary expansion possibly due to gene duplication “Fig 6”. Further, the divergence of CHS genes into many isoforms results in the formation of CHS multigene family which combats the demand for flavonoid biosynthesis under stressful environmental conditions. In most of the angiosperms, CHS has been found as a multigene family, such as in petunia (violet 30) [59], morning glories (Ipomoea) [60], Gerbera [52], leguminous plants [24], and *Cannabis sativa* [61]. Functional validation of CHS genes from different species reveal broad demarcation in both regulation and function among duplicate CHS genes. Gene duplication event, a widespread feature of plant genomes is measured to be key mechanism for evolutionary advances and functional divergence [49,50]. Under uncritical gene dosage conditions, it is tenable that one copy may defend the other during natural selection. One copy retains the original function and the other may attain a novel function favoured by natural selection [62].

The differential transcript levels of *Ga*CHSs in different organs was in agreement with the earlier studies on CHS multigene family of *Glycine max* and *Gerbera hybrida* [57,63]. The variable expression patterns indicate the efficient selection of *Ga*CHS isoforms. The discrepancy in temporal and spatial expression patterns of *Ga*CHS isoforms advocates their significant differentiation in developmental regulation in the plant. Among the two isoforms, *Ga*CHS2 was highly expressed in reproductive tissues than *Ga*CHS1 which displayed its expression in vegetative plant parts. *Ga*CHS2 expression was highest in male part (stamens) of the flower than in other parts suggesting its possible role in pollen maturation where it may be expressed with a strict, temporal and spatial regulation at the transcriptional level. Similar anther expression patterns were reported for other anther specific CHSLK cDNAs from *B*. *napus* BA42 [64], *O*. *sativa* YY2 [65], *S*. *latifolia* CCSL6 [66]. Moreover, the biochemical diversity in plants under different environmental conditions upholds several copies of CHS genes which are expressed at different developmental stages in various tissues [12]. Some isoforms are constitutively expressed, while others are induced by different environmental stresses [67]. The diverse expression patterns of CHS reflect its wide role in plants [68].

Flavonoid accumulation in different vegetative and reproductive tissues corroborated well with the differential *Ga*CHS expression. The significance of CHS expression and flavonoid accumulation for the formation of functional pollen has been demonstrated by several workers on CHS mutants [69,70]. Transgenic plants suppressed for gene encoding chalcone synthase (CHS), were instrumental in identifying such an essential role of flavonols in pollen function. In *Arabidopsis*, LAP5 and LAP6 encode anther-specific proteins with homology to CHS and play an important role in the synthesis of pollen fatty acids and phenolics found in exine. Mutations in either gene result in abnormal exine patterning, whereas the lap5 lap6 double mutant produces pollen grains devoid of exine, causing strong male sterility [71].

Characterization of the CHS multigene family in order to get insight into more CHS members other than *Ga*CHS1 and *Ga*CHS2 in *G*. *asiatica* needs further investigation. The *In Planta* functions and substrates are yet to be determined. The role of these proteins in metabolite accumulation and plant development needs further validation. Also, exploring the role of *Ga*CHS2 in pollen specific flavonoid biosynthesis and pollen fertility maintenance in *G*. *asiatica* needs added attention. As we have earlier reported the establishment of an efficient *in vitro* multiplication and genetic transformation system for *G*. *asiatica* [72], which could be deployed for the molecular interventions for the development of commercially acceptable seedless cultivar(s) in *G*. *asiatica*by tinkering with specific *Ga*CHS. The goal can be achieved by using advanced biotechnological interventions like CRISPR/cas system. This system has been useful to edit the genomes of major crops such as rice [73], wheat [74], tomato [75], soybean [76] and potato [77]. Further, CRISPR/cas system is better gene editing technique than RNAi due to off-target effects of the latter.

## Conclusion

Characterization and determining the functional efficacy of CHS isoforms is important for specific aims like disruption of male function for the induction of parthenocarpy. Towards this objective, present study entails cloning and characterization of two isoforms of *Ga*CHSs from *G*. *asiatica*. Further, enzyme kinetic studies and their substrate selectivities confirm their enzymatic potential. Expression analysis of *Ga*CHs showed that *Ga*CHS2 isoform is maximally expressed at anthesis stage and at this stage, it is copiously expressed in male part of the flower (stamen). These empirical findings are suggestive of *Ga*CHS2 being a possible gene target for impairing male reproductive function by using advanced molecular tools like CRISPR/cas system as a future strategy.

## Supporting information

S1 Fig Conserved residue prediction for *Ga*CHS1 and *Ga*CHS2. Conserved residue analysis of *Ga*CHS1 and *Ga*CHS2 were performed using ConSurf and ConSeq web servers. Residue conservation from variable to conserved is shown in blue (1) to purple (9). Abbreviations: e = exposed residue according to the neural-network algorithm; b = buried residue according to the neural-network algorithm; f = predicted functional residue (highly conserved and exposed); s = predicted structural residue (highly conserved and buried); and X = insufficient data, the calculation for this site was performed on less than 10% of the sequences.
(TIF)

S2 Fig SDS-PAGE profile of expressed recombinant proteins. Time-course expression of *Ga*CHS with different concentrations of IPTG (0.6 mM, 0.8 mM and 1.0 mM). The cultures were harvested at different time intervals (4h, 6h and 8h) and the collected samples were analysed on 10% SDS-PAGE.
(TIF)

S3 Fig SDS-PAGE profile of purified recombinant proteins. SDS-PAGE (10%) of affinity purified recombinant proteins from *E*. *coli* BL21 (DE3) cells transformed with pGEX-*Ga*CHS1 and pGEX-*Ga*CHS2 expression cassettes. Lane 1, standard protein marker; Lane 2, purified recombinant GST-fused *Ga*CHS1 protein; Lane 3, purified *Ga*CHS1 protein after removal of GST; Lane 4, purified recombinant GST-fused *Ga*CHS2 protein; Lane 3, purified *Ga*CHS2 protein after removal of GST.
(TIF)

S4 Fig Kinetic study of *Ga*CHSs. **(A-B)**; Michaelis-Menten plots of *Ga*CHS1 (A) and *Ga*CHS2 (B). The kinetic parameters K_m_ and V_max_ were calculated by nonlinear regression analysis using GraphPad Prism 6 software. The values for K_m_ and V_max_ in *Ga*CHS1 and *Ga*CHS2 were 46.49±1.94, 47.02±6.82 and 35.06±2.48, 123.02±19.30 respectively.
(TIF)
