# Supplementary figures and images for: Molecular and functional characterization of two isoforms of chalcone synthase and their expression analysis in relation to flavonoid constituents in Grewia asiatica L
Source: PLoS One. 2017 Jun 29;12(6):e0179155. doi: 10.1371/journal.pone.0179155 (PMC5491003; doi:10.1371/journal.pone.0179155)

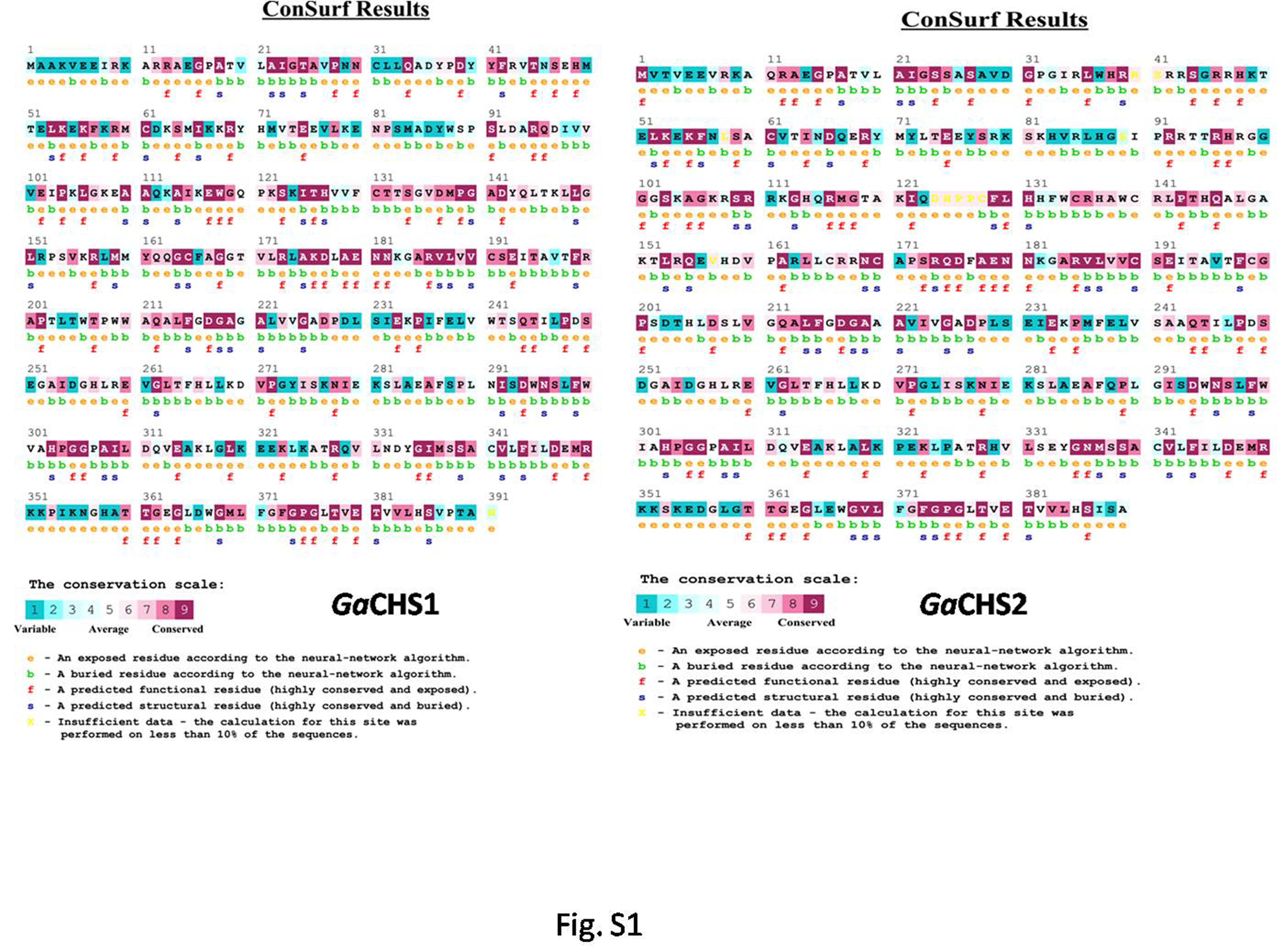

Supplement: S1 Fig — Conserved residue analysis of GaCHS1 and GaCHS2 were performed using ConSurf and ConSeq web servers. Residue conservation from variable to conserved is shown in blue (1) to purple (9). Abbreviations: e = exposed residue according to the neural-network algorithm; b = buried residue according to the neural-network algorithm; f = predicted functional residue (highly conserved and exposed); s = predicted structural residue (highly conserved and buried); and X = insufficient data, the calculation for this site was performed on less than 10% of the sequences. (TIF) [file pone.0179155.s001.tif]

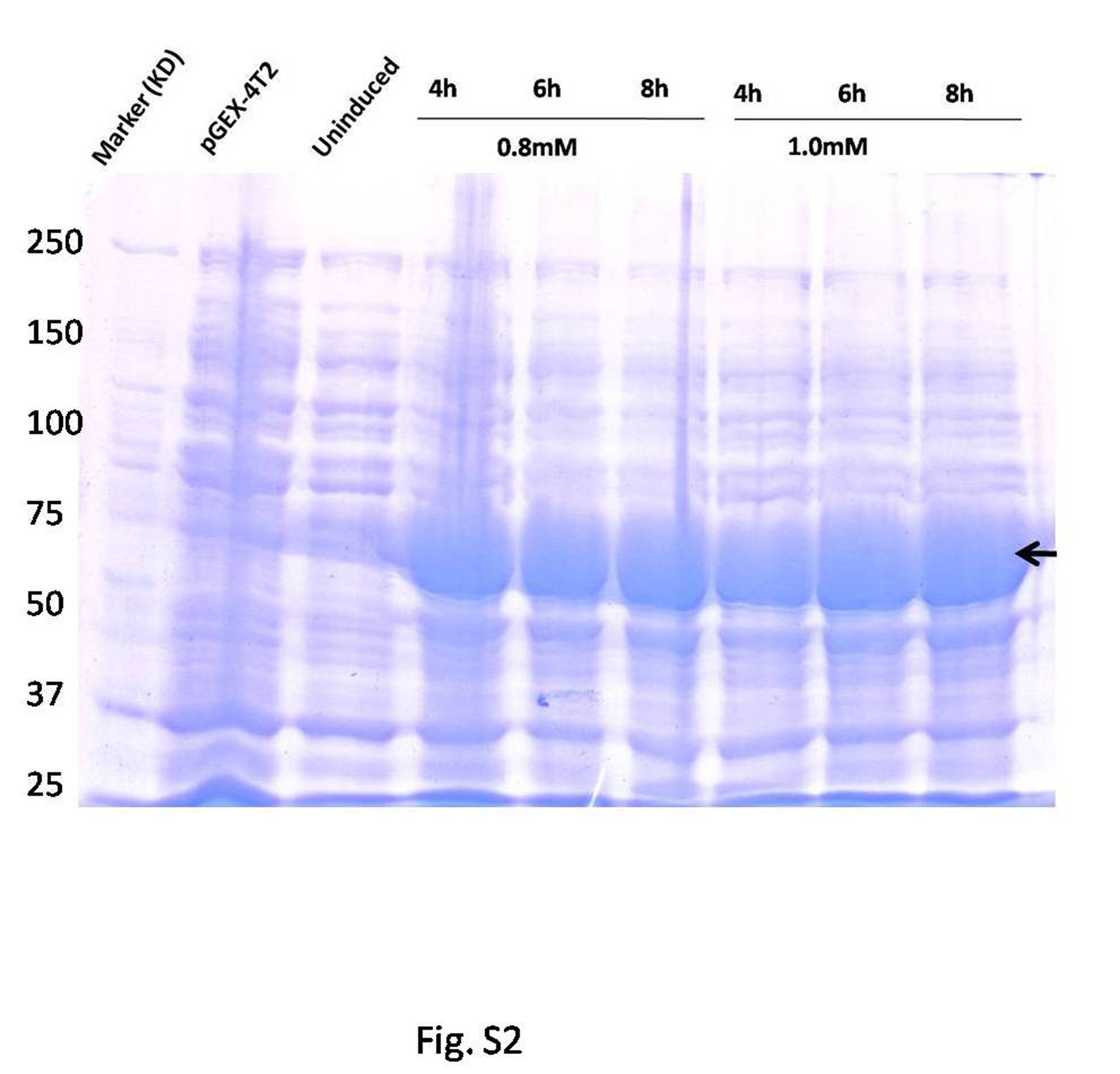

Supplement: S2 Fig — Time-course expression of GaCHS with different concentrations of IPTG (0.6 mM, 0.8 mM and 1.0 mM). The cultures were harvested at different time intervals (4h, 6h and 8h) and the collected samples were analysed on 10% SDS-PAGE. (TIF) [file pone.0179155.s002.tif]

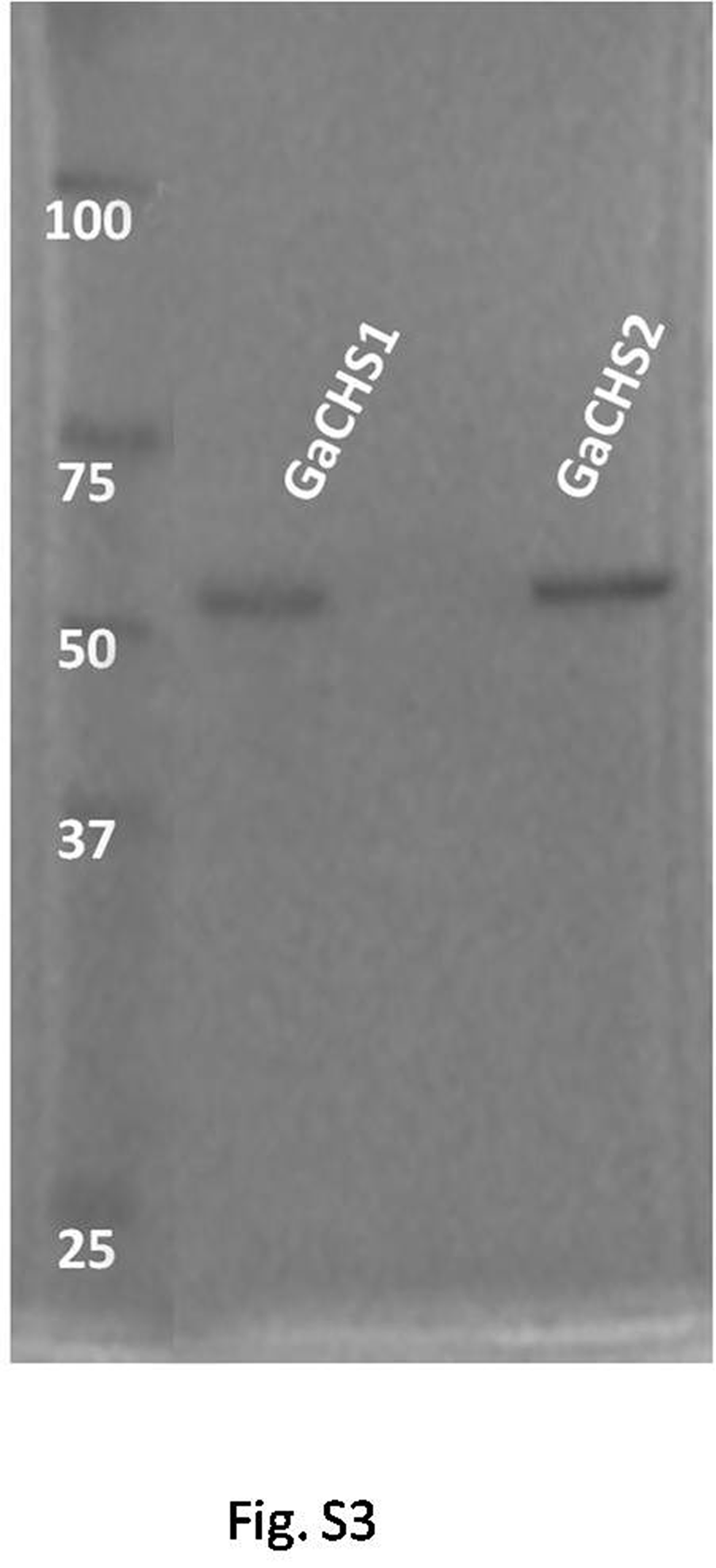

Supplement: S3 Fig — SDS-PAGE (10%) of affinity purified recombinant proteins from E. coli BL21 (DE3) cells transformed with pGEX-GaCHS1 and pGEX-GaCHS2 expression cassettes. Lane 1, standard protein marker; Lane 2, purified recombinant GST-fused GaCHS1 protein; Lane 3, purified GaCHS1 protein after removal of GST; Lane 4, purified recombinant GST-fused GaCHS2 protein; Lane 3, purified GaCHS2 protein after removal of GST. (TIF) [file pone.0179155.s003.tif]

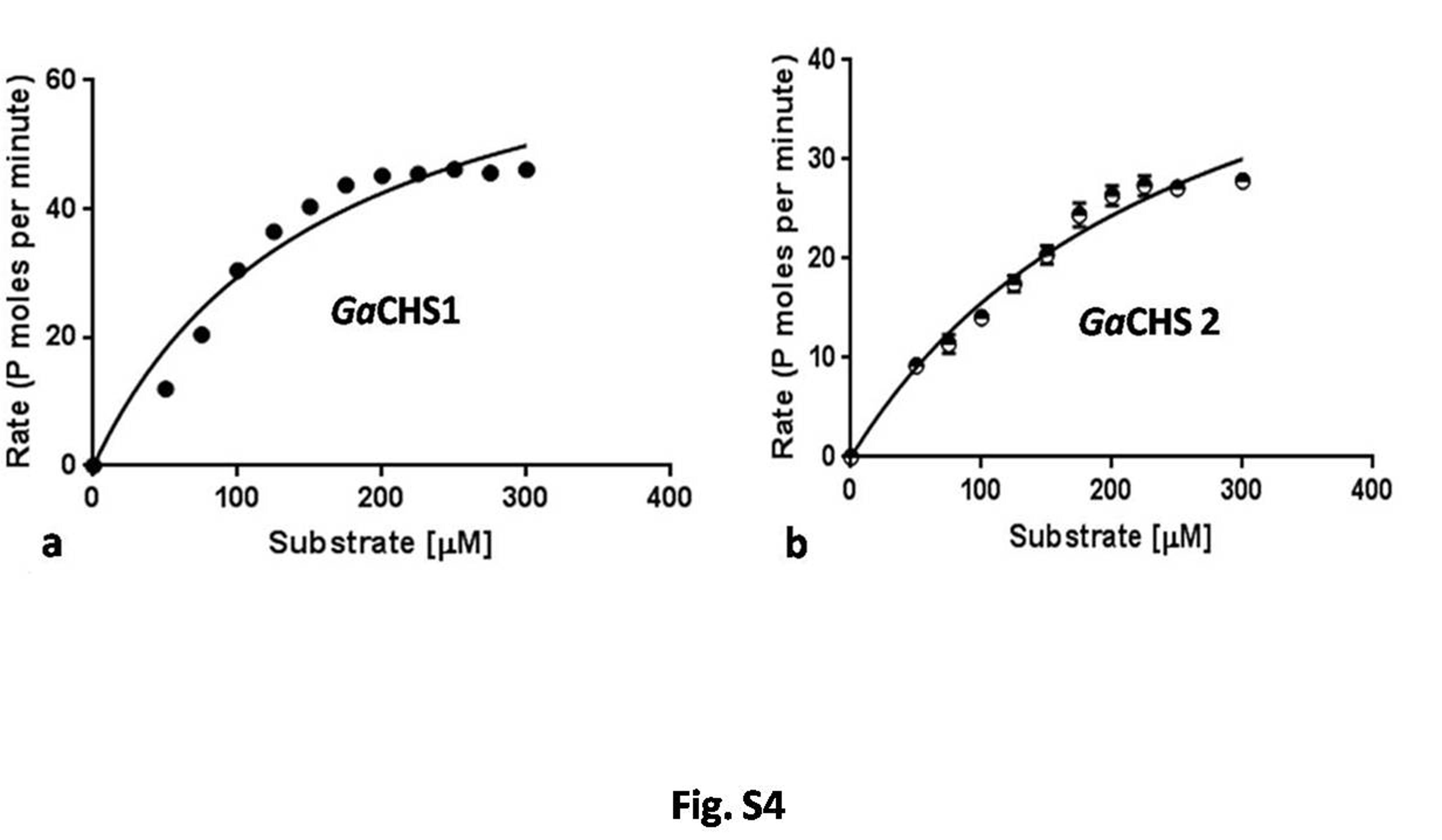

Supplement: S4 Fig — (A-B); Michaelis-Menten plots of GaCHS1 (A) and GaCHS2 (B). The kinetic parameters Km and Vmax were calculated by nonlinear regression analysis using GraphPad Prism 6 software. The values for Km and Vmax in GaCHS1 and GaCHS2 were 46.49±1.94, 47.02±6.82 and 35.06±2.48, 123.02±19.30 respectively. (TIF) [file pone.0179155.s004.tif]
